# *Micromégas*: Altered Body–Environment Scaling in Literary Fiction

**DOI:** 10.3389/fpsyg.2016.00556

**Published:** 2016-04-21

**Authors:** Sebastian Dieguez

**Affiliations:** Laboratory for Cognitive and Neurological Sciences, Département de Médecine, Université de FribourgFribourg, Switzerland

**Keywords:** literature, embodiment, architecture, cognition, bodily consciousness

## Abstract

Architectonic embodiment postulates a bidirectional link between bodily awareness and the architectural environment. The standard size and features of the human body, for instance, are thought to influence the structure of interiors and buildings, as well as their perception and appreciation. Whereas architectural practice and theory, the visual arts and more recently the cognitive sciences have explored this relationship of humans with their crafted environments, many fictional literary works have long experimented with alterations of body–environment scaling. This so-called Gulliver theme – popular in the science-fiction genre but also in children’s literature and philosophical satire – reveals, as a recurrent thought-experiment, our preoccupation with proportions and our fascination for the infinitely small and large. Here I provide an overview of the altered scaling theme in literature, including classics such as Voltaire’s *Micromégas*, Swift’s *Gulliver’s Travels*, Caroll’s *Alice*, and Matheson’s *The Shrinking man*, closely examining issues relevant to architectonic embodiment such as: bodily, perceptual, cognitive, affective, and social changes related to alterations in body size relative to people, objects and architectural environments. I next provide a taxonomy of the Gulliver theme and highlight its main psychological features, and then proceed to review relevant work from cognitive science. Although fictional alterations of body-environment scaling far outreach current possibilities in experimental research, I argue that the peripetiae and morals outlined in the literary realm, as products of the human imagination, provide a unique window into the folk-psychology of body and space.

For I am the size of what I see not my height’s size.

Fernando Pessoa (*A Little Larger Than the Entire Universe: Selected Poems*)

I want to walk like a giant on the land

Neil Young

## Introduction

The human body has long been used as the main provider of measure units. The thumb, the hand, the arm, the foot, as well as the step and the throw, through centuries, have helped humans scale their natural and architectural environments, in a way that made immediate functional and psychological sense to them (Tuan, 1977; Emmons, 2005). Likewise, objects themselves are provided with “arms,” “handlers,” and “legs,” and “bodies” are found in the vast cosmos as well as in the infinitely small. More generally, spaces and places are unconsciously represented in relation to our body: “The human being, by his mere presence, imposes a schema on space. Most of the time he is not aware of it.” (Tuan, 1977, p. 36). Upright and prone, high and low, central and peripheral, left and right, front and back, small and large, inside and outside, far and close, here and there, are all concepts that are meaningful only through bodily mappings. “In a literal sense, the human body is the measure of direction, location and distance (…) Spatial prepositions are necessarily anthropocentric” (Tuan, 1977, pp. 44–45).

Similar ideas have struck the imagination at least since Protagoras (c. 490 – c. 420 BC) famously stated that “man is the measure of all things.” Although the exact context to that statement has not reached us, it is usually interpreted as a relativistic and humanist manifesto: “reality” is merely how the world appears to each of us, and man is at the center of all things. From this general insight, it should come as no surprise that architects have long sought to elucidate the relationship between, on the one hand, the morphology and capacities of the human body and, on the other hand, how humans perceive and appreciate their built environments. This bidirectional linkage of bodily awareness and crafted environments, prominent in Renaissance architectural thinking, is currently studied under the name “architectonic embodiment” (see Pasqualini et al., 2013, and references therein). Whereas “early explanations of scale show that empathetic bodily projection is critical to imagining a future edifice” (Emmons, 2005, p. 232), architectural structures and alterations of one’s environment can in turn modulate human bodily, emotional and cognitive experience (Tuan, 1977; Joyce and Verpooten, 2013).

While the study of architectonic embodiment foremost needs integrating the expertise of a number of fields such as architecture, engineering, computer science, urban sociology, demography, biology and cognitive science, I would like to suggest here that it could also benefit from another, perhaps unlikely, source: literary fiction. Indeed, one difficulty for developing a science of architectonic embodiment is the sheer inconvenience, or impossibility, of manipulating material variables such as the human body or its immediate environment. But we can *imagine* such alterations, as well as their consequences. This is not only what scientists and architects have to do anyway as part of their jobs, but also what writers have done routinely since the advents of the epic, the drama, the tale, and the novel. They have asked what would happen if man or the world were suddenly to dramatically change, and they have provided fictional answers, through their imaginary character’s experiences, perceptions, and thinking. By doing so, across time, their proposals have in turn sometimes made it into common knowledge, and thus entered a wider imagination beyond their own’s and their readership’s. I have argued elsewhere that literary contributions, far from mere ramblings of unconstrained imaginations, often reflect, and provide insight on, the very structure and organization of human cognition as well as its dysfunctions. This holds also, and indeed especially, for pure works of fantasy, including science-fiction and fairy-tales, where the world, characters and events depicted by the writer cannot stretch farther than what their reader’s brains can digest. In this sense, it does not matter whether fiction is “scientifically accurate” or not. Just like major inaccuracies and errors in the visual arts can provide powerful insights about the normal workings of the visual system (Cavanagh, 2005), errors, exaggerations, and plain inventions in literary and cinematic fiction can be much informative about cognition in general. What matters is whether, how and why works of fiction manage to tap into the relevant cognitive mechanisms that allow a story to be understood, enjoyed and remembered, and possibly become widely successful on the cognitive market. On this view, I have argued that stories depicting amnesiac characters, although often neurologically inaccurate, provide a very rich corpus of folk conceptions of memory, highlight its central link to identity and culture, and enrich our understanding of the history of memory and amnesia research (Dieguez and Annoni, 2013b). More relevant to the present topic, I have reviewed a large set of literary writings and clinical and experimental research on the theme of “the double,” underscoring the major connections existing between so-called “autoscopic phenomena” and the “Doppelgänger” of gothic and horror stories, as well as recent trends in cognitive neuroscience focusing on the mechanisms of bodily awareness, which all strongly suggest a common reliance on neurocognitive processes dealing with how humans represent their own body and its place in space (Dieguez, 2013). It is important to note that the claim here is not that “writers knew it all along” or that they have somehow “predated” later scientific discoveries. Rather, and more simply, writers, readers, patients, and scientists, as well as architects and, for that matter, everybody else, share a similarly organized brain allowing them to successfully imagine, understand, investigate, and communicate ideas about the human mind, based on a necessarily limited set of cognitive processes relevant to, or even specialized for, the topic at hand.

With this caveat in mind, the present contribution sets out to pursue this approach with an eye on architectonic embodiment. If man truly is “the measure of all things,” then surely dramatic alterations of the body or the environment would alter that “measure.” It turns out that writers have long explored this idea through the “popular motif of a change in scale” (Spiegel, 2008, p. 377), introducing straightforward alterations in scale in their stories. Interestingly, this very “scalar fascination” (Emmons, 2005, p. 229) has been linked to that of architects: “Like an architect making a drawing, these stories describe a person venturing into an imaginary world of another size” (Emmons, 2005, p. 229). I first provide an overview of this surprisingly rich literature, and then examine its parallels with recent “size perturbation paradigms” (Karok and Newport, 2010) and related approaches in cognitive science. Although the disconnect between both domains (literary and scientific) is currently Gargantuan, it is hoped that a concomitant awareness of both fiction and research could help scientists and architects alike shrinking that gap to Lilliputian dimensions.

## The “Gulliver Theme” in Literature

### The Gulliver Theme

**FIGURE 1:**
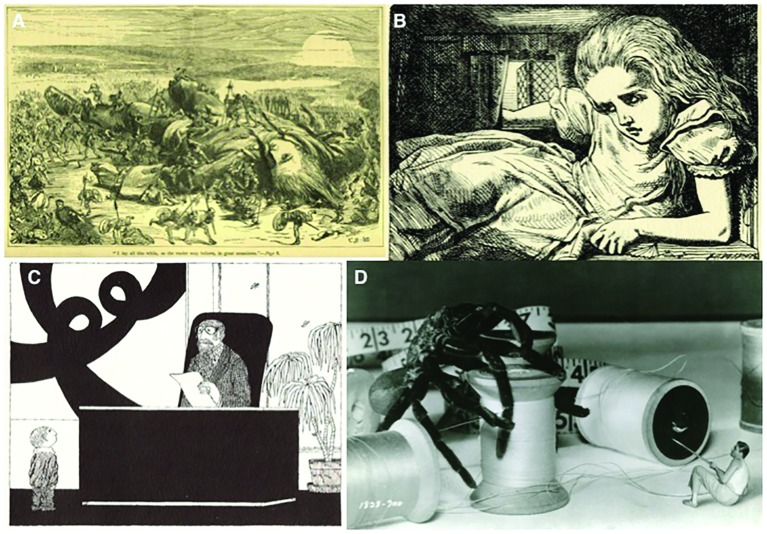
**Examples of the Gulliver theme in fiction, spanning political satire, children’s tales, and science-fiction/horror. (A)** The iconic discovery of the Lilliput people in Swift’s *Gulliver’s Travels* (illustration: Thomas Morten, 1865). **(B)** Alice’s fourth scale alteration in *Alice’s Adventures in Wonderland* (illustration by John Tenniel, 1866). **(C)** In *The Shrinking of Treehorn* by Florence Parry Heide (1971; illustration: Edward Gorey), the progressive shrinking of a child protagonist illustrates feelings of insignificance and the scaling disparity that separates the adult and child worlds. **(D)** In *The Incredible Shrinking Man* (Jack Arnold, 1957), adapted from Richard Matheson’s *The Shrinking Man*, the protagonist fights for his life using commonplace items as weapons.

Fiction has long provided an imaginative field to explore alterations of body–environment scaling, be it as philosophical musings, satirical thought-experiments, children tales, or science-fiction stories (**Figure 1**). French writer and literary critic Messac (1936) probably provided the first (and only) full monograph on the topic, entitling it after Voltaire’s tale *Micromégas^1^.* Complaining of his overwork as a schoolteacher and of his “children cries in too small an accommodation,” Messac lamented having to publish his impressive review in an incomplete form, but felt confident that “it does not seem that anyone would care to do better.” While listing and commenting on about a 100 authors and works relevant to the issue, he unfortunately failed to define it properly and, initiating a long lineage of scientifically minded critics, focused mostly on the physical and biological impossibility of altering the scales of the universe as well as producing giant or microscopic humans, animals, or insects. Schuhl (1952) provided a similar and shorter treatment of the topic, naming it “the Gulliver (or Micromégas) complex.” This literary theme, he wrote, is “one of the favorite illusions of our imagination, which believes it can shrink or enlarge the beings around us at will, to the stature of giants or bugs, with no further alterations” and reflects something that “most spontaneously haunts our thoughts: the theme of the Large and the Small.” I propose that the terminology “Gulliver theme” can be safely adopted, but I’d rather define it as follows: the Gulliver theme encompasses all literary treatments highlighting, as a central part of the plot, dramatic and fantastical departures from the commonly known biological, material, and cultural human body–environment scaling. In what follows I review, roughly in the chronological order, the many ways authors have exploited the Gulliver theme highlighting Voltaire’s *Micromégas*, Swift’s *Gulliver’s Travels*, Carroll’s *Alice* as well as examples from children’s literature and the science-fiction genre. This review is not meant to be exhaustive, but suffices to provide a broad overview of principles, functions, effects, and mechanisms underlying the Gulliver theme, which will then be examined in light of cognitive science research in order to delineate the many insights literature can offer the science of architectonic embodiment.

### Micromégas

The above definition would include stories of mythological giants such as the Olympian deities, Ulysses fighting Polyphemus the Cyclop, David defeating Goliath, Pygmies climbing over Heracles’ body, fairies and Hobbits, Tom Thumb and *Le Petit Poucet*, Rabelais’ *Gargantua* and *Pantagruel*, and many more. The appeal of such characters very often lies in their striking size-disparity toward other characters and the environment: indeed, “the coming together of two figures of different scales is enough to produce a startling impression” (Schuhl, 1952, p. 64). Voltaire’s (1752) *Micromégas* exaggerates this feature to the extreme. The main character, coming from the star Sirius, is said to be about 24’000 times taller than an average man and stands, according to the fanciful measures provided by Voltaire, somewhere between 24 and 32 km tall. He is accompanied in his travels through the cosmos by a Saturnian about a 20th of his size, making him a dwarf to him, but still an impressive figure to the humans they end up meeting (“atoms” to them) when they reach earth. Of course, the story allows putting into perspective the conceit of human philosophers, the insignificance of earthly quarrels and the fundamental limits of scientific knowledge. At the same time, the giants are impressed by the intelligence of these “invisible insects” and the story also serves as a philosophical warning against holding prejudices against the unknown and the dangers of judging people by their appearances or their status. *Micromégas*, however, probably pushed the satire too far. The characters are so ridiculously large that the imagination is excessively boggled and simply cannot cognitively digest their adventures (e.g., mountains are “pinky grains” to Micromégas, yet he manages to pick a ship full of humans and “put” it in his hand). Perhaps this was intended, as the “Gulliver theme,” quite aside from specific meanings and lessons, often purports to convey a vertiginous sense of the infinite and a disturbing awareness of the limits of our senses (Schuhl, 1952). This sense of disquiet and awe for the infinitely large and the infinitesimally small has been famously captured by Blaise Pascal: “For in fact, what is man in nature? A nothing in comparison with the Infinite, an All in comparison with the Nothing, a mean between nothing and everything” (quoted in Monk, 1955, p. 57). The French philosopher likewise brought attention to a further development, namely the then widespread idea of interlocking worlds of different scales: what bugs are to us, Malebranche, among others, suggested, some other animal must be to them, and so forth infinitely. The idea was not lost on Gulliver himself: “Undoubtedly Philosophers are in the right when they tell us, that nothing is great or little otherwise than by Comparison. It might have pleased Fortune to let the *Lilliputians* find some Nation, where the People were as diminutive with respect to them, as they were to me.” Ultimately, the idea implies that the infinitely small and large are one and the same reality, as suggested by numerous myths and tales (e.g., Paris, 1875; Bachelard, 1957). This idea of multiple scales populating the universe has been speculated as being very likely associated to the inventions of the telescope and especially the microscope (Nicolson, 1935; Stewart, 1993; Emmons, 2005), who brought to the human eye for the first time, as it were, entirely new worlds to explore and in the process diminished the importance and centeredness of human’s place in the universe, ideas likewise prominently featured in stories of the Gulliver theme.

### Gulliver’s Travels

Voltaire’s allegory was inspired by, and even quotes, Jonathan Swift’s (1726) *Gulliver’s Travels* (**Figure 1A**), a work much better remembered perhaps, in part, because the altered body–environment scaling stays within manageable metrics. In the first two parts of Lemuel Gulliver’s memoir, the narrator discovers successively two countries where everything, including people, is scaled such as he is a giant (in Lilliput) or a dwarf (in Brobdingnag). According to Swift’s indications, the countries are, respectively, scaled 1:12 and 12:1 to Gulliver’s size, including people, animals, trees, buildings, and objects. This literary device allows for maximal satirical effects and much humorous inventions, yet it is clear that Swift attempts to convey some sense of plausibility (not least by having Gulliver repeatedly state that he aims to stay truthful to his observations) and very carefully exploits the change of perspective induced in and across both worlds. Although Gulliver’s actual body size stays constant – underscoring his status as an “average good man” (Monk, 1955, p. 55) – it stands to reason that his experiences, actions and perceptions would dramatically differ in Lilliput and Brobdingnag, and this is masterfully executed despite numerous inaccuracies in both worlds scaling that have baﬄed critics (Brady, 1978; Rabb, 2013). However, along with the striking effect of altering body–environment scaling in both directions, Swift, as a satirist, might well have intentionally chosen to somewhat exaggerate his character’s tallness in Lilliput and his “littleness” in Brobdingnag, in order to create slightly unpredictable worlds and a sense of estrangement, as well as making an ironic hint as to the unreliability of his narrator (he is indeed telling a “tall tale”: in his words, “a severe Critic would be apt to think I enlarged a little”) and the satirical nature of his book. It makes no sense otherwise that Gulliver can hold six Lilliputians in his right hand, and much less that his handkerchief can be used as an exercise field for 24 Lilliputian horses (our purported tolerance to size/scale errors notwithstanding, see Rabb, 2013). Nevertheless, comparisons of different scales are constant in the book. In Brobdingnag, the mind-boggling scale of Gulliver’s new environment drives him, much more than in Lilliput, to draw comparisons from monumental structures of his original environment (namely, normal-sized England), not without some difficulties: “I reckoned our Coach to be about a Square of *Westminster Hall*, but not altogether so high; however, I cannot be very exact.” Gulliver thus draws from memory to provide a meaningful metric for him, as his ongoing body–environment scaling is otherwise too difficult to comprehend. In Brobdingnag, for instance, he fully realizes that the inhabitants look to him as he himself looked to the Lilliputians, providing awareness and self-realization through forced social comparisons.

As the book deals mainly with human politics and its failings, size is used in the first two parts as a method to investigate the nature of power and chauvinistic pride: “Swift’s attack on pride in the first two voyages is made more powerful because of his brilliant use of the chain of being. In so far as, we recognize ourselves in the Lilliputians or in Gulliver in Brobdingnag, we become aware of our pettiness, of the disproportion of our race and of the shocking difference between what we profess and what we are” (Monk, 1955, p. 60). Gulliver’s general attitude dramatically changes in both countries: In Lilliput he is kind and considerate, in Brobdingnag he is forced to be careful and courageous (Monk, 1955). As Gulliver utterly dominates the Lilliputians, he feels safe and confident among them, and readily submits to their pretentious rulings in order to better study them. Even though they call him “the Man-Mountain,” Lilliputians actually feel superior to Gulliver and are not afraid of him (indeed, they think they have captured him). Altering body–environment scaling allows bringing to the fore the ridiculous conceit of some men and structures of power, unable to realize their own insignificance and complete lack of leverage when claiming to deal with “large” issues. To drive his point across, Swift has Gulliver simply urinate over the royal palace to extinguish a fire, a deed for which he would be accused of treason, at which point he simply leaves the country. In Brobdingnag, things are different. He is immediately terrified by the gigantic inhabitants, which, however, mean him no harm but strive to exploit him as an amusing and sensationalistic attraction. Gulliver then becomes abjectly insignificant, first as a freak among the hoi polloi, then a “plaything” for the girls, ladies, and queen of the high society. Whereas in Lilliput he could “seize” anyone at will if needed, in Brobdingnag he is the one that is a mere affordance to others. He must shout to make himself heard, is moved around in a small box arranged with dollhouse equipment, has to suffer through the bullying of a Brobdingnagian dwarf and must constantly fend off countless dangers (rats, flies, wasps, a frog, a monkey, abysmal heights, crushing weights, formidable sounds, and winds…). Poorly adapted to their current environment, his senses are constantly offended by overwhelming stimulations. Disgust is a recurrent feeling for Gulliver during his 2 years stay in Brobdingnag, especially in relation to the magnified skins, smells, faces, and breasts of the country’s females, as well as the huge amounts of food they ingurgitate in his presence (Monk, 1955). Issues of status and power often interact with questions of masculinity, sexuality, and gender differences in the Gulliver theme (Monk, 1955; Armintor, 2007; Rabb, 2013), foremost for Gulliver himself, who alternates between play-doll and sex-toy among the feminine hands that own him: “[The] most uneasiness among these maids of honor (…) was to see them use me without any matter of ceremony, like a creature who had no sort of consequence.”

Perhaps the most important feature of *Gulliver Travel’s* is the *defamiliarization* effect it achieves on nearly every page. This process of “cognitive estrangement” and “de-automatization of perception” involves the twofold mechanism of naturalizing the strange and making the familiar strange: “whenever a marvelous element is introduced into a seemingly realistic world, a collision occurs between two systems of reality, producing an estranging effect. The familiar appears in new surroundings and is thereby re contextualized” (Spiegel, 2008, p. 375; see also “the alienation of the familiar” in Sobchack, 1997). Swift is tireless when it comes to describing common objects as gigantic structures or miniatures, in and across his imaginary worlds: a cow can be smuggled into one’s coat-pocket, a bowl of cream suddenly becomes a dangerous pool, golden coins become worthless specks, a golden ring becomes a hunky mass of riches. What seemed familiar and unnoticeable in a standard context is made, by altering the body–environment scaling, exceptional, incomprehensible, and remarkable. New types of usage and interaction emerge, making in turn the unfamiliar familiar again: in general, characters embroiled in the Gulliver theme strive to adapt. Another important insight from *Gulliver’s Travel* worth emphasizing here is the introduction of a size-after-effect, whereby the subject mixes up different scales. This is altogether absent from the voyage to Lilliput: while Gulliver needs some time to realize he has perceived a “real boat” at sea, namely a boat scaled to his actual size (and looking like a “prodigious vessel” to the people of Blefuscu, who are scaled like their neighbors of Lilliput), he is not in the least impressed by its size, and when he first encounters people normally scaled to his size upon returning to England, he is not surprised either by their size at all. Upon returning home from Brobdingnag, however, he is struck by the visual size of the first Englishmen he meets at sea: “I was (…) confounded at the Sight of so many Pygmies, for such I took them to be, after having so long accustomed mine Eyes to the monstrous Objects I had left” (149). We also learn that the effect existed during his stay: “while I was in [Brobdingnag], I could never endure to look in a Glass after my Eyes had been accustomed to such prodigious Objects, because the Comparison gave me so despicable a Conceit of myself.” Interestingly, looking at himself in a mirror induced one of the only self-size illusion in the book: “I really began to imagine myself dwindled many Degrees below my usual Size.” Even back at home in England, and within his family, the effect strongly persists: “observing the Littleness of the Houses, the Trees, the Cattle, and the People, I began to think myself in *Lilliput*”; “My wife ran out to embrace me, but I stooped lower than her knees, thinking she could otherwise never be able to reach my mouth. My daughter kneeled to ask my blessing, but I could not see her till she arose, having been so long used to stand with my head and eyes erect to above sixty foot.” These seemingly proportional and longstanding after-effects, Gulliver ascribes to “an Instance of the great Power of Habit and Prejudice.” Finally, *Gulliver’s Travel* also points to a technical scaling problem Swift had to deal with, that of the transition between different worlds. Where do Lilliput, England and Brobdingnag start and end? Some amusing (perhaps unintended) inconsistencies appear when Gulliver stands on the boundary of the discovered lands. In Lilliput’s sea, he can “wade” several 100 m before he is “forced to swim,” either making the sea a Lilliputian one, or Gulliver himself *de facto* a giant in a normal-sized sea. Brobdingnag’s sea seems different, as “Sea-fish are of the same Size with those in *Europe*, and consequently not worth catching” for the giant people. However, “a Hailstone is near eighteen hundred times as large as one in *Europe*.” Why such discrepancy? Gulliver merely “leave[s] the Reasons to be determined by Philosophers.” Seeing gigantic eagles from Brobdingnag fly away in the distance, normal-sized human sailors remark “nothing of their being larger than the usual Size,” which Gulliver supposes “must be imputed to the great Height they were at.” This is, surprisingly, one of the rare comments on the phenomenon of size-constancy I was able to find in the Gulliver theme literature.

### Alice

Up to now, I have dealt with alterations of body–environment scaling that did not involve alterations of the bodily self *per se*. Humans under the lens of Micromégas, and Gulliver throughout his travels, keep their usual human size. This was still the case when the Gulliver theme entered the horror and science-fiction genre, with James Fitz-O’Brian’s (1858) *The Diamond Lens*. In that short-story, an outcast fascinated by microscopy learns from Leeuwenhoek’s spirit^2^ how to devise a super microscope. Using it, he discovers in a drop of water an entirely new world, populated by a feminine creature whom he calls Animula and immediately falls in love with. Animula never realizes she is being observed, and the lover is condemned to watch her dry away. This tale highlights the paradoxical distance of neighboring worlds, when scales are dramatically different: “forgetful for an instant of everything save her presence, I withdrew my eye from the microscope eagerly, – alas! (…) my gaze fell (…) on a colorless drop of water. There (…), this beautiful being was forever imprisoned. The planet Neptune was not more distant from me than she,” “I would have gladly forfeited all claims to my human birthright, if I could only have been dwarfed to the size of an animalcule.” As Messac notes, up until the publication of *The Diamond Lens*, the small and the large where so to speak fixed, authors imagined no way to travel from one scale to the other. He notes that Wells’ (1904) *The Food of the* Gods will be the first major work to introduce the possibility of altering characters’ physical size at will (using special pills), but he curiously omits a much better known book, namely *Alice’s adventures in Wonderland* (**Figure 1B**) by Lewis Carroll (1866). “Little Alice” indeed is frequently confronted to sudden changes in her bodily size and/or in her environment (which is, after all, a “Wonderland”). The Gulliver theme, then, reaches here its maximal expansion: not only can the environment’s scale be diminished or enlarged (as in *Gulliver’s Travels*), but at the same time Alice’s body can do the same. Soon after she falls “down a large rabbit-hole,” she finds herself in a room where the only escape is “a small passage, not much larger than a rat-hole.” While she wonders how she could possibly “shut up like a telescope,” she finds a bottle marked with the words “DRINK ME,” and after obliging she finds herself shrinking down to “ten inches high”: “she was now the right size for going through the little door.” After eating a piece of cake marked “EAT ME,” she then finds herself growing until “her head struck against the roof,” at “more than nine feet high.” Twelve occurrences of such changes occur during the story, either following consumption of some item or spontaneously, most often at convenient moments when there is a need for a change of scale to pursue the adventure, and sometimes offering alternative solutions: “if it makes me grow larger, I can reach the key; and if it makes me grow smaller, I can creep under the door: so either way I’ll get into the garden, and I don’t care which happens!” At moments, Alice gets confused and doesn’t remember whether she is tall, small or normal, for instance when she is swimming in the pool of her own tears and mistakes a mouse for “a walrus or hippopotamus.” In fact, along her adventures she seems to have lost track of her original size: “I must have been changed several times since then (…) being so many different sizes in a day is confusing.” Alice’s experience of body–environment scaling alterations have usually been interpreted as an embodied metaphor of children’s preoccupation with size, body, identity, and growth (Schuhl, 1952; Kibel, 1974; Hunt, 1995; MacArthur, 2004). While at first her small size and sudden magnifications make her feel insecure, defenseless and depersonalized (“Who in the world am I?”), she progressively gains confidence and control, stops crying and eventually finds a dominant stature during the absurd trial near the end of her dream, where the characters suddenly acquire their normal scale as she grows, and thus a more familiar meaning: ““Who cares for you?” (…) (she had grown to her full size by this time). “You’re nothing but a pack of cards!.”” “Growing up” entails conflicting notions for a child. The constant injunctions to behave like an adult or a “grown-up” conflict with the child’s actual status and size, which necessarily confine her in a subordinate and vulnerable state. And yet, the process is ineluctable and irreversible, a fact Lewis Carroll was painfully aware of as he much deplored the loss of innocence and imagination of the adult world, and apparently wished that her beloved little Alice would never grow up. By embodying the contrast and arbitrariness of child-adult scales in his fiction through the Gulliver theme, Carroll produced an immortal allegory encompassing children’s imagination, anticipations and anxieties, as well as adults’ memories of a long-forgotten, gigantic and amazingly rich world.

### Children’s Literature

Unsurprisingly, well before and after *Alice*, the Gulliver theme has featured prominently in folktales and literature aimed at children. Tales collected and written by the Grimm brothers, Charles Perrault, Hans Christian Andersen, and others include stories of diminutive heroes, dangerous ogres and giants, plots unfolding in dollhouses, worlds within worlds, as well as magical transformations of the body, objects or the environment. Perhaps the simple (biological and cultural) facts that children are smaller than adults, that growth is a unidirectional process, and that human environments are by and large adult-scaled worlds, suffice to explain the appeal of the miniature for children. Yet the miniature and the gigantic also imbue the adult imagination and feature in a wide array of cultural facts and items (Stewart, 1993), suggesting powerful psychological and social factors involved in the production, appreciation and maintenance of the Gulliver theme across centuries. In her study of the “miniature hero metaphor” in children’s literature, Hunt (1995) highlights “three versions of the miniature,” namely the “solitary dwarf,” the “miniature society,” and the “shrinking character.” E. B. White’s (1945) *Stuart Little* embodies the solitary dwarf motif. A two inches tall child-mouse, lost in New York City could predictably embody the feelings of helplessness, insecurity, insignificance and neglect experienced by children in the adult world. Small equals unimportant. Yet the protagonist is smart, courageous, and resourceful. The reader (or listener) of the story thus feels empowered by Stuart Little’s engaging behavior and attitude despite his smallness, and has an empathic response to him because he or she knows what it feels like to be aware of one’s value while being ignored, derided or infantilized by the grown-ups. Furthermore, the fact that Stuart Little is a mouse in a world of humans adds to the sense of otherness and incompleteness, as well as to the bodily preoccupations of growing children achieved by the mere alteration in size (in *Alice*, this mechanism is reversed as it is the other characters that have non-human features). The main use of the Gulliver theme here is thus to induce a sense of wish-fulfillment toward more independence, control and power for the “little one’s.”

Mary Norton’s (1953) *The Borrowers* serves to illustrate the motif of the “miniature society” or “small world.” In this series, a clandestine species of small creatures cohabits secretly with humans and interact with the human-scaled world. Stories of the large and the small societies thus run in parallel and sometimes intersect, and the audience can chose to identify with the little or the normal world. Although they are despised by humans, the Borrowers can exert a great appeal to children: they live in their own secret world, they have to be smart and creative in order to hide and survive, and they frequently triumph over human adults. Perhaps there is also a strong appeal for the type of society the Borrowers have built, the epitome of the miniature as “a metaphor for the interior space and time of the bourgeois subject” (Stewart, 1993, p. xii). Indeed, the Borrowers universe is cozy, cute, homogeneous, safe, familial and hierarchically organized. Ordinary human-scaled objects are used as elements of furniture and architecture (a cigar box is a bed, stamps are pictures on the wall, blotting paper becomes a rug…). In a nutshell (as it were), they live in a dollhouse, and they spy on the normal world. The motif here is that of control, as the small world created is one that feels comfortable and is socially manageable.

*The Shrinking of Treehorn*, by Florence Parry Heide (1971; **Figure 1C**) illustrates the shrinking character theme. Although a little boy suddenly starts growing small, nobody, including his parents, takes notice. The theme here is neglect, the scourge of not being attended to or not being taken seriously. As is the case for a number of “adult” shrinking stories, the phenomenon starts with the character’s clothes suddenly becoming too large (highlighting how altered body–environment scaling, from the perspective of the self, can be noticed only by comparison with stable-sized surrounding artifacts or people). And just like its science-fiction counterparts (see below), the theme introduces the fragility of self-worth, fantasies of destruction, disappearance and annihilation, questions about origins and goals, preoccupations with bodily appearances and experiences, and the “Idea of marginalization” (Hunt, 1995, p. 130): “Books about shrinking (…) retain their fascination; they pose questions which matter to child and adult alike. The matter of one’s origin and eventual dissolution underlie questions of control over one’s body, societal response, and changing perceptions. These books deal with the inescapability of change, the unreliability of perception (both the hero’s perception of others and theirs of him or her), and the unpalatable fact that life has a beginning and, thus, an ending. Like the other varieties of the miniature hero metaphor, this one satisfies a widely felt need among young readers; unlike the other two, its fascination does not recede in adulthood” (Hunt, 1995, pp. 133–134).

### Science-Fiction

Turning to fantasy and science-fiction treatments of the Gulliver theme – indeed a prominent and “traditional sf theme” (Evans, 1994, p. 386) –, we find a variety of devices used to shrink and/or enlarge people, animals, and objects^3^. The device can be a wish granted by a fairy in Han Ryner’s (1901) *L’Homme-Fourmi*; a disease induced by radioactivity coupled with an insecticide in Richard Matheson’s (1956) *The Shrinking Man*; chemical compounds in Wells’ (1904) *The Food of the Gods*, Ray Cummings’ (1923) *The Girl in the golden atom*, and Maurice Renard’s (1928) *Un Homme chez les microbes*; some “energy” produced in a human-sized glass jar in René Spitz’s (1938) *L’Homme élastique* (which introduces the twist that characters can grow small or large, while keeping their original mass) and A. Bleunard’s (1893) *Toujours plus petits* (in the latter, the “energy” turns out to be mere hypnotic suggestion); a highly sophisticated labroom in Isaac Asimov’s (1966) *Fantastic Voyage* (adapted from the Richard Fleischer’s movie that appeared the same year, 1966); or some unexplained curse as in Jean-Charles Rémy’s (1976) *L’Arborescence* and Mark Wersinger’s (1947) *La chute dans le néant*.

Dominating in this genre is the micro use of the Gulliver theme. Except for *The Food of the Gods* and *L’Arborescence* where characters unidirectionally grow large, science-fiction usually prefers shrinking protagonists or plot devices that allow “growing” in both directions. A notable exception, however, is the famous cinematic horror-trope of the “attack” of giant creatures such as ants, reptiles, spiders, and men or women, but in these cases it is usually the hero characters that are made to look small in contrast. While these movies often reflect the nuclear and Cold War anxieties of the times, offer a comment on the power and abuses of science, technology and the military, and have been taken to allude to Freudian ideas as well as the topics of gender, race, paranoia, and invasion, the sheer entertainment value of gigantic size as also been noted (inducing awe and fear), as well as the satirical value of artificially overturning the world order by putting humans at the bottom of the food chain, and thus drawing attention to their destructive powers in the real world. These effects are all the more efficient as bugs are almost natural phobic stimuli – and thus “superoptimal” stimuli when they cover large portions of the visual field -, and they also are both extremely familiar and alien to humans, enhancing the “cognitive estrangement” effect in their gigantic form (Sobchack, 1997; Berton, 2006; Leskosky, 2007; Tsutsui, 2007).

The “reversal of perspective” trope is brought home particularly well in a cinematic example: the episode *The Invaders* [written by Richard Matheson (1961)] from *The Twilight Zone* series tells the story of a woman harassed by minuscule alien visitors. After much trouble, when she finally manages to destroy their minuscule spaceship, we read on its side that it belongs to the “US Army,” its equipage just managing to warn (in English) their base to stay clear from this planet, as it is inhabited by gigantic and dangerous beings.

Another recurrent trope is that of the “interior voyage,” where diminished characters are made to visit the inside of the human body, often to great didactic effects and creating feelings of wonder. Alfred Taylor Schofielden’s (1887) *Travels in the interior*, Edwin Pallander’s (1902) *The Adventures of a Micro-Man*, and Mark Twain’s (1905) *Three Thousand Years among the Microbes* are early illustrations of this literary device (see Brodesco, 2011 for contemporary examples). Visits in the ant-world have also long been a specific and prominent sub-theme of the Gulliver theme, perhaps starting with Emer de Vattel’s (1756) *Les Fourmis* (see also Messac, 1936 and Vas-Deyres, 2013). These two tropes reveal our fascination with the human body and its secrets, and situate ants as the epitome of the small autonomous society, perhaps the most obvious “world inside the world” for observant humans. Together, they highlight one paradoxical feature of the Gulliver theme: the tendency to anthropomorphize alter-scaled worlds. The “change in perspective” is thus frequently minimal, as compared, for instance, to *Gulliver’s Travels*: smaller worlds in many books and movies are essentially the same as the human-scaled world in terms of social and cognitive features.

Along with many features already reported above (summarized in the next section), science-fiction’s usage of the Gulliver theme adds, as expected, many elements derived from the sciences, such as considerations on the interaction of gravitational and surface tension forces, the physics of the shrinking process (do atoms themselves shrink, or is the distance between them reduced?), the issue of the relationship between sense data and altered sense organs (how would diminished retinas and brains capture and interpret unaltered photons?), and, more interestingly for the field of architectonic embodiment, the altered experience of time during body–environment scaling alterations (see e.g., *The Girl in the Golden Atom*, *Un Homme chez les Microbes* and *Fantastic Voyage*). However, science-fiction is also concerned about the interconnections of scientific concepts and social concerns. In that respect, Richard Matheson’s (1956) *The Shrinking Man* (brought to screen a year later by Jack Arnold as *The Incredible Shrinking Man*; **Figure 1D**) probably remains the most complete and successful treatment of the Gulliver theme. Staying in the same environment – the suburban American home – the ineluctable shrinking process of Scott Carey, the main protagonist, allows for many dramatic effects and powerful metaphors. These include, through an astutely constructed narrative (and remarkable special effects for the movie), careful explorations of bodily and perceptual alterations, feelings of estrangement toward a progressively growing spatial environment (the same objects acquire different meanings as the shrinking insidiously proceeds; distances and actions suddenly become insuperable from one page to the other), commentaries on the consumer culture, importance of social status, as well as gender roles of the 1950s, changes in attitude and character of the protagonist (from childish outbursts at the beginning, passing through paranoid and depressive states as he becomes an outcast, to full survival mode in the final sequence, it has frequently been noted that Carey’s character grows mentally and humanely as his physical dwindling proceeds), the need for constantly adapting in an increasingly unpredictable, dangerous, and unfamiliar world, alterations of the experienced passage of time, and of course metaphysical questions about the infinite and the unknown, as well as the inevitability of self-annihilation (Rosenheim, 1995; Sobchack, 1997; Hendershot, 1998; Berton, 2006; Cunnally, 2013).

## Taxonomy and Overview of the Gulliver Theme

“Writers of the microscopic” (Evans, 1994, p. 394), ever since Swift and Voltaire, have often had to deal with problematic issues of scaling, proportionality and verisimilitude. Leaving aside the physical and biological consequences that would ensue from straightforwardly altering body–environment scaling^4^, the difficulties of the constraint are well paid off by the many creative inventions and insights it allows. Using the “technique of the impossible” (Schuhl, 1952, p. 13) uniquely afforded by the literary art, writers have covered a broad range of the landscape provided by our capacity for “imaginative size” (Jessup, 1950). I have explored selected works exploiting the Gulliver theme, by genre, author, and more or less in chronological order. A much precise analysis would be needed to note the very frequent cross-references between these works, which highlights the fact that most of the features described here can be found across the whole Gulliver theme specter, and especially to consider the linguistic and narrative devices exploited to create the sense of an altered body–environment scaling, notably through metaphors, comparisons, numbers, and metric units, as well as lexicons pertaining to the body and space. Here, I keep the focus large and propose a tentative taxonomy of the Gulliver theme, as well as a summary of its features most relevant to architectonic embodiment.

### Taxonomy of the Gulliver Theme

**FIGURE 2:**
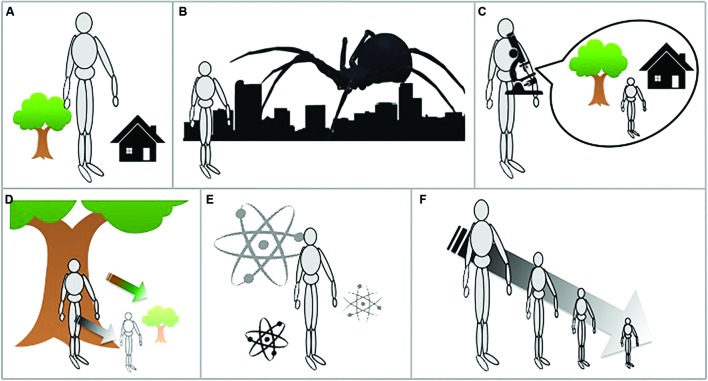
**Taxonomy of the Gulliver theme. (A)** The self keeps its regular size, only the rest of the environment is size-altered (e.g., *Micromégas*, *Gulliver’s Travels*); **(B)** The self keeps its regular size, only selected “characters” alter their size (usually insects or spiders, but also humans, e.g., in *The Food of the Gods*); **(C)** Another scale is observed from a distance (e.g., via a super microscope in *The Diamond Lens* and *The Girl in the Golden Atom*); **(D)** Both the body and the environment are suddenly size-altered (e.g., *Alice in Wonderland*); **(E)** Another scale (the subatomic world, the human body^5^, an anthill) is visited through sudden or almost immediate self-scaling alteration, either through some device or involuntarily (e.g., *The Girl in the Golden Atom*, *L’Homme-fourmi*, *Fantastic Voyage*); **(F)** The bodily size-alteration is progressively and insidiously experienced (e.g., *The Shrinking Man*, *L’Arborescence*).

Any alteration of body–environment scaling mechanically produces a twofold process: the small induces feelings of bigness, the large induces feelings of smallness. Which is to say that as the human body, as experienced from the first-person (egocentric) perspective, shrinks, the entire environment is concomitantly perceived as larger than normal, and vice-versa. This taxonomy centers on the subjective first-person perspective of body–environment altered scaling, usually that shared by a main character and its empathetic reader, and does not distinguish the direction of the change (all possibilities from the microscopically small to the unbelievably large seem to have been exploited). Six plot devices, I think, suffice to encompass the whole range of the Gulliver theme, and many works involve more than one of them (**Figure 2**).

### Features of the Gulliver Theme

In my review of the Gulliver theme in literature, I have highlighted a number of features, or literary tropes, that seem to cut through the above taxonomy and repeatedly appear across different genres and times. These features help explain the functions and appeal of the Gulliver theme, and provide the starting point for a cognitive and experimental approach bridging the gap between fictional and real life body–environment interactions. What follows is an overview of these main features, spanning a considerable range of human experiences.

### Perception

The visual modality is prominent in the Gulliver theme: objects, scenes and people suddenly or progressively loom enormous, or dwindle into miniatures, whatever the process involved. A common literary method is to compare proportions of size-altered items with the normal (remembered) metric of corresponding or other familiar items. Authors also like to play with measure units: these are either from the normal-sized world (e.g., “one meter” is thus an enormous distance for a shrunken human), or are scaled to the current altered bodily size (e.g., the top of a flower or a table is said to be several hundred meters high). At times the scales can intermix, leading to much confusing, striking, or amusing situations or descriptions. Characters frequently pass through a process of adaptation or habituation to the new environment, which involves confusions about scales and size after-effects as the body–environment scaling alterations progress or reverse. Authors have also exploited other modalities: sounds, smells, and tactile sensations are minimized, magnified, or transformed altogether as a character’s size, or that of the environment, change scales. For instance, a giant has to lay low and pay attention to hear Lilliputian people, a giant’s perfume is offensive to a normal-sized nose, an even ground becomes a rocky and perilous area to a microscopic character. Alterations of body–environment scaling thus lead to numerous multisensory effects. Less explored are the effects on the experience of time, which would necessarily accompany any dramatic changes in space and distances.

### Action and Bodily Awareness

Like Gulliver, Alice’s experience of body–environment scaling alterations brings to the fore the psychological notion of “affordances.” Following Gibson (1979) and Proffitt and Linkenauger (2013) write that an affordance reflects “the functional utility of objects and surfaces for an organism having a particular body and behavioral repertoire. For example, a rock affords being picked up if it is of a size that can be grasped (…) With respect to Gulliver and the Lilliputians, the affordances of a given rock vary greatly depending on the actor. What Gulliver sees as a pebble is a substantial boulder to the Lilliputians.” Distances, heights, volumes, and sizes thus reflect the morphology and action repertoire of intentional agents, a notion, as the previous quote exemplifies, exquisitely captured by the fictional Gulliver theme. Likewise, the cognitive *Umwelt* that is afforded to agents varying in size, most notably to human children, is a frequent topic explored by the Gulliver theme, with special emphasis on the universal biological fact of growth. Fictional alterations of body–environment scaling provide a unique means to develop, think about and gain insight into this issue.

### Cognition, Emotions, and Attitudes

More prominently than the previous features, the Gulliver theme has put much focus on the affective and behavioral consequences of body–environment alterations, thereby anticipating current interest for the notion of “embodiment” in cognitive science (e.g., Gibbs, 2006). Here the notions introduced are wide-ranging. Central to the Gulliver theme is the idea of “estrangement” of the familiar, or sudden awareness that many normal, usual or trivial aspects of ordinary life can be seen from completely novel perspectives provided changes in modes of body–environment interactions. Emotionally, the most frequent reactions are negative: as the environment becomes larger than usual, characters experience disorientation, fear, a sense of impending danger, and even paranoia and disgust toward things, animals, and people. The feeling of awe is perhaps an exception, with protagonists sometimes marveling at their rediscovery of the world from new perspectives. Social cognition is likewise much affected in the Gulliver theme: characters go through enhanced episodes of social comparison, and the themes of dominance, submission, and isolation take on a most prominent form as characters either grow large or small. Indeed, the Gulliver theme allows for philosophical musings about human awareness of the Other (differences are seen in a new light through changes in perspective/scaling, as in Heraclitus’ analogy: “Man is called a baby by god, even as a child by a man”), the relativity of, and possibility to alter, one’s value systems, as well as about the limits of human perception and knowledge.

I suggest that this non-exhaustive proposal for a taxonomy and effects of alterations of body–environment scaling could be well-suited as a starting point for a wide-ranging research program in architectonic embodiment. I provide such an overview in the next section, keeping in sight the previous literary contributions, which after all are the products of the same human imagination shared by writers and scientists alike. Altering the scales of human reality is undoubtedly one of the most straightforward and simple ways to disrupt ordinary experience, and thereby reveals our implicit reliance and expectations about the structure of space, places, people and things, as well as our place therein. Making the “normal” world unpredictable, or less predictable, is a central *modus operandi* of fantastic and horror fiction (Dufour, 2006), as well as experimental research.

## The Cognitive Psychology of Body–Environment Scaling

The Gulliver theme has been addressed by literary, philosophical, and cultural studies (Schuhl, 1952; Bachelard, 1957; Stewart, 1993), physics and biology (Haldane, 1926; Messac, 1936; Moog, 1948), aesthetics (Jessup, 1950), history of science (Nicolson, 1935), cinema and science-fiction studies (Tsutsui, 2007; Spiegel, 2008), architecture (Emmons, 2005), political theory and urbanism (Rey, 2014) and feminism (Berton, 2006; Armintor, 2007; Cunnally, 2013). While all these approaches have provided worthwhile, if rather scattered and partial contributions to the Gulliver theme, what has been missing, to the best of my knowledge, is a cognitive approach to the topic.

Psychological research directly relevant to the Gulliver theme is scarce, but many scattered observations and findings in different domains and processing levels provide insight into the possible mechanisms involved in body–environment scaling. In what follows I hint at selected examples organized along the major themes presented in the previous section, namely perception, action, cognition, emotion, and general attitudes/beliefs.

### Perception, Action, and Bodily Awareness

Visual scientists have long studied the mechanisms, development, disorders, and neural correlates of size perception, often exploiting specific visual illusions and examples from natural phenomena and perspective in the visual arts. Most notably, the phenomenon of size-constancy, or “phenomenal regression to the “real” object,” whereby changes in distance do not alter perception of an object’s size, has afforded much insights and experimental paradigms (see e.g., Ross and Plug, 1998; Dieguez et al., 2011). However, in recent years ecological approaches to perception have been revived, reinstalling bodily and motor processes at the center of the general perceptual mechanisms allowing size perception and estimation. Indeed, our spatially situated and embodied experience of the world is now standardly considered as contributing to perceptual processes as much as purely visual mechanisms, including our ability to mentally represent, transform and rotate material objects (e.g., Tarr and Pinker, 1989). Proffitt and Linkenauger (2013), for instance, have argued that basic biological bodily metrics, such as eye height or arm length, as well as physiological processes and behavioral goals and preferences, shape the way we perceive distances and sizes, the logic being that these concepts are intrinsically linked to our potential for acting in the world (see review in Proffitt and Linkenauger, 2013). The concept of *Action boundary*, for instance, asserts that the perceptual world is scaled to our body, and specifically to those aspects or parts of the body that are directly relevant and available to one’s goals. Empirical evidence has supported this approach. In a series of experiments, Linkenauger et al. (2010) have shown that the dominant hand provides a stable metric to scale graspable and manipulatable objects, as demonstrated by the combined findings that altering the visual size of the dominant hand (through magnifying or minifying glasses) modifies verbal and motor measures of size estimation for objects (see also Karok and Newport, 2010; Bernardi et al., 2013), and that the perception of the size of the dominant hand itself is resistant to visual size-alterations, as compared to one’s feet, other person’s body parts, or familiar objects (Linkenauger et al., 2014).

Such experimental “tricks,” the laboratory’s best approximation of the Gulliver theme, have also been extended from body parts to the entire body, using multimodal (mostly visuo-tactile) techniques to create the sense of embodying differently scaled bodies, or more sophisticated virtual reality equipments. Thus, van der Hoort et al. (2011) and van der Hoort and Ehrsson (2014) could induce in participants the vivid sensation of inhabiting a small (a Barbie doll) or a huge manikin, as assessed through questionnaire and physiological measures. Importantly, when such manikins were successfully embodied, through concurrent tactile and visual stimulations of the real and the virtual bodies, participants over- and under-estimated the size of objects and walking distances, as their illusory body shrunk or expanded, respectively.

Other modalities (e.g., auditory and olfactory) have yet to be explored with such paradigms. An older study, however, provided evidence that phenomenological time is associated with body–environment scaling. Subjects were asked to imagine themselves as a scale figure in a reduced model-environment. During successful immersion, participants estimated the felt passage of time to be proportional to the scale they were immersed in. In a 1/12 model (similar to that experienced by Gulliver in Lilliput), participants estimated a duration of 30 min (in the real world) to be elapsed after about 2.5 to 2.9 min in the reduced environment, a timing strikingly close to the 1/12 spatial ratio (DeLong, 1981; see also Gorea and Hau, 2013).

Some clinical conditions also provide insight into the Gulliver theme. Neurologists have even dubbed some peculiar symptoms of migraine or epilepsy “Alice in Wonderland syndrome,” which refers to distortions of body–environment scaling following disturbances of the somatosensory cortices or the posterior parietal cortex. In these cases, patients experience body parts or their entire body to be disproportionately large or small, which also affects the perception of the size of objects, people and the environment. Such sensations are sometimes also called micro- and macrosomatognosia when they affect the body, and micro- and macropsia when they affect the visual system, mostly after disturbances of the occipital lobes. Some hallucinations, foremost observed as part of Charles Bonnet syndrome, are called “Lilliputian,” and refer to the illusory perception of small characters (for reviews of altered states of bodily awareness, including size illusions, see Dieguez and Blanke, 2011; Dieguez and Annoni, 2013a).

### Cognition, Emotions, and Attitudes

Although the perception of size continues to be a major issue for visual science, the issue pervades many other domains of cognitive, affective, and social psychology. Words can convey strong inferences and representations about body–environment scaling. Priming studies have shown how mental representations of objects and animals implicitly carry with them information about their referents’ physical size, which in turn can influence judgments and comparisons with other items and different domains of magnitude, such as time, speed, pitch, numerosity, or even luminosity.

Among many such studies, Gabay et al. (2013) found that flashing a picture of an animal influences a subsequent parity detection task in a systematic way: conceptually larger animals (e.g., an elephant) facilitated detection of parity for larger digits (e.g., 8,9), whereas conceptually smaller animals (e.g., a cat) facilitated detection of parity for smaller digits (e.g., 2,3). Likewise, other studies (e.g., Rubinstein and Henik, 2002) have shown that the physical size of the stimuli themselves (words, digits, pictures) influences numerical cognition, suggesting that numerosity and size perception share a common core processing system, perhaps a magnitude processor. Indeed, this line of research indicates that human neonates (de Hevia et al., 2014) and 9 months old infants (Lourenco and Longo, 2010) spontaneously deal with size, numerosity and duration as features of a general magnitude representation system. If a stimulus mapped into a feature such as large size, this same stimulus was also expected by preverbal infants to be more numerous and to last longer.

Such findings suggest a strong sensitivity to size and proportions, not only at the perceptual and motor level, but more generally in how humans represent and make sense of the world. In this regard, the notion of “psychological distance,” as developed through “construal-level theory” (Liberman and Trope, 2008), posits that distance from the spatially and temporally situated self (in the “here and now”), be it physical, temporal, social, or conceptual distance, is associated to higher- and lower-level mental construals. Object, people, or ideas that are farther away from our immediate experience are represented in an abstract, schematic and decontextualized fashion, whereas those that are closer are represented more concretely, in detail and in context. In turn, representations that are abstract are experienced as farther away than those that are concrete, which are felt as closer. “Distance,” thus, perhaps as coded through a general and all-purpose system of magnitude representation, leads to predictable effects on how we represent the world around us and in our minds.

Although research is lacking in this respect, it is possible that such approaches are constrained by the body–environment scaling that is typical of our species, and thus metrics that far bypass the capacity for human reach and navigation, be it physical or imaginary, would require other types of cognitive processes (Proffitt and Linkenauger, 2013; Rips, 2013). I suggest that the Gulliver theme is one of these processes. Indeed, the capacity for fictional body–environment scaling alteration seems to have a functional value. DeLoach et al. (1997) tested 2 1/2 years-old children in a search task: the young participants were shown an object in an experimental room and then asked to retrieve its counterpart miniature in its corresponding place in a scaled-down model of the room. It was found that miniature retrieval was facilitated when children were lead to believe that the real-size room had been shrunken thanks to a “shrinking machine,” and thus that the original room and the model were in fact one and the same. Thus, not only did young children apparently accept the science-fiction scenario as real, but the findings suggest that the capacity for mentally altering scales is acquired and mastered earlier than the dual representation necessary for symbolically processing the very concept of a model. Perhaps this natural ability for mentalizing changes helps explain the popularity and easiness to process fictional altered-scales, as well as its very existence as a literary and cinematic trope.

How we perceive and represent body–environment scaling has also strong effects on the emotions. One striking example is the human fascination with dwarfs and giants, which not only pervades mythology but is instantiated in the cultural history of so-called “freaks.” Spectacular representations of dwarfism and gigantism, notwithstanding their sometimes morbid nature, reflect the normative value of familiar bodily metrics. Tellingly, in show-business promotional postcards, these individuals were frequently positioned next to familiarly sized objects or people, “to emphasize the contrast in size” (Enderle, 1998). Such emotional sensitivity to bodily size contrasts might be related to the normal and slow biological process of growth, which not only has been personally experienced by each of us, but is also observable in our own children. Thus, the so-called “baby illusion” refers to the parents’ impression that their child suddenly appears bigger when a new baby is born in the family. This was reported by over 70% of mothers surveyed and it was experimentally demonstrated that the effect resides in an underestimation of the youngest-children height (by about 7.5 cm in average, regardless of his/her exact age), whereas estimates of the elder-child remained basically accurate (Kaufman et al., 2013). Such perceptual exaggeration of the youngest-children smallness might have a functional value, in that it would enforce and stabilize care for the most needful child. That the effect is a sudden rather than gradual “defamiliarization” feeling is also a strong indicator that intricate and robust perceptual, cognitive, affective, and behavioral components govern the psychology of size.

A well-documented effect of size pertains to the relationship of power with verticality, which might be related to the previous considerations on growth. As Schwartz et al. (1982) put it: “the vertical classification system carries this symbolic burden because it is rooted in a general feature of human development, namely, the experiential analog between social inequality and the statural inequality of child and parent.” Priming studies have thus shown that we spontaneously associate the dimensions “high” or “up” with dominance and power, an effect that has also been documented outside of the lab. Stulp et al. (2015), for instance, have observed that taller individuals display dominant interpersonal behavior during brief dyadic interactions: they are for instance less likely to alter their path or give way in busy streets. Height has also been shown to influence political popularity and the outcome of elections (Stulp et al., 2013), as well as myriad other social factors (Herpin, 2006), perhaps suggesting an evolutionary advantage to form coalitions with “physically formidable” individuals (Murray, 2014).

Experimentally altering participants’ sensed bodily size through immersion in a virtual body and a virtual environment, in addition to modulating object-size and distance estimation (see above), has also been shown to alter emotional and social experience. Introducing the so-called “Proteus effect,” Yee and Bailenson (2007) have found that interacting through an avatar 10 cm taller than a virtual confederate, participants displayed more confident and tough behavior in a negotiation task than when they were assigned a 10 cm shorter avatar. In a better controlled study, Banakou et al. (2013) found that adult participants immersed in a 4 years-old toddler’s body estimated virtual objects as larger than when immersed in a scaled-down adult avatar of the same size, an effect that was only present when ownership of the avatar’s body was successfully induced (namely, when its movements were synchronous with those of the participant’s physical body). Additionally, participants in the child body ownership condition associated their own self-concept to that of childhood, as demonstrated through reaction times in an implicit association task. A more specific emotional change through virtual altered scaling was reported in a study by Freeman et al. (2014). Women with paranoid tendencies participated in a virtual reality study. In the shorter condition, in which they inhabited an avatar 25 cm shorter than their real height, they reported more negative social comparison ratings and higher paranoid ideation feelings, with respect to virtual passengers of a subway ride. The effect of lower-height on paranoia was entirely mediated by the social comparison scores, indicating that stature is indeed a marker of self-worth, dominance, confidence, desirability, and sociability (the lack of which could lead to negative intention-attributions toward others). As noted in previous sections, such cognitive, emotional, and attitudinal changes following altered body–environment scaling have been widely exploited in works such as *Alice in Wonderland*, *Gulliver’s Travels*, and *The Shrinking Man*. To mention an additional and striking nod to the latter work – where the considerably shrunken protagonist fights a black widow in his cellar – a study by Vasey et al. (2012) found a link between estimated spider size (participants were exposed to actual tarantulas) and spider phobia: aversion to spiders resulted in spider-size overestimation. Perhaps such an effect is exploited by the many “giant bug movies” of post-war American science fiction.

### Beliefs and Values

More profound effects of size and height disparities relative to the human body have been suggested, pointing to possible relationships of architectonic embodiment with belief systems. In a theoretical paper, Joyce and Verpooten (2013) proposed that across cultures and history, monumental religious architecture exploited human’s sensitivity to size-related clues. Pursuing a dual Darwinian approach, they interpret very large buildings for religious purposes as costly signals and the result of sensory exploitation. Briefly, monumental religious architecture, through its sheer wastefulness, provides a “non-ambiguous and reliable signal of power” while at the same time tapping into an “adaptive sensitivity for bigness.” Combined, these mechanisms provide a powerful vehicle for cultural transmission. Because the sheer vastness of massive edifices is attention-grabbing and overwhelms the senses and thought, it instills shared notions of prestige and feelings of awe in individuals, who, in turn, perceive themselves as submissive and insignificant (Shiota et al., 2007). Thus religious monumental edifices might function as devices enforcing social stratification and adherence to the belief system associated with them. Additionally, massive buildings might favor openness to supernatural beliefs through the very awe they induce, and provide a context for social activities and rituals, thus making them especially suited for religious purposes.

Although speculative, such an account illustrates the far ranging ramifications of body–environment scaling for an architectonic embodiment research program. Notably, the experience of awe provides a promising locus for interfacing perceptual systems and higher-level value construals. A similar idea has permeated philosophical thinking since Aristotle’s concept of “aesthetic size” (Jessup, 1950) and received its first physiological account in Burke’s (1757) *Philosophical Enquiry into the Origin of Our Ideas of the Sublime and Beautiful*. Burke stated that it “is too evident, and the observation too common” that “Greatness of dimension is a powerful cause of the sublime” (Part II, Section 7). To explain this phenomenon, he suggested that large stimuli are composed of “a vast number of distinct points, every one of which, or the ray from every one, makes an impression on the retina. So that, though the image of one point should cause but a small tension of this membrane, another and another, and another stroke, must in their progress cause a very great one, until it arrives at last to the highest degree; and the whole capacity of the eye, vibrating in all its parts, must approach near to the nature of what causes pain, and consequently must produce an idea of the sublime” (Part IV, Section 9). Of course, notwithstanding its dubious merits, such a proposed visual mechanism would not account for the metaphorical uses of vastness in other modalities or in writing (viz. Darwin’s closing statements in *The Origin of species*: “There is *grandeur* in this view of life”), and for the literary examples reviewed in this paper [see also Bachelard’s (1957) extended analysis of the use of the word “vast” in Baudelaire’s poetry and essays].

Other tentative links between size cognition and belief systems, values and spirituality accrue, for example, from reports of large unidentified marine animals sightings which, despite inadequate evidence, have been widespread since the 18th century and are not explained by sighting distance (Paxton, 2009). Such probable hoaxes and legends again point to our fascination and sensitivity with extraordinary body–environment size discrepancies, which striking character seems to favor their very popularity and memorability. At the individual level, as suggested in *Gulliver’s Travels* and other literary works reviewed herein, values might also be altered by consideration of our relative size. Suedfeld et al. (2010) examined 125 astronaut autobiographies and found that upon returning to Earth from a spaceflight, astronauts displayed increased concern with values oriented toward the collective good, such as universalism and spirituality. That such changes might partially be linked to altered size-environment scaling is suggested by astronauts’ testimonies reprinted in the paper: “Maybe if people had a chance to see this [the Earth from space], they wouldn’t be so parochial, they wouldn’t be so interested in their own particular territories. (…) To me and I think to all of us, it was a realization that our world is finite, it is small, it is fragile, and we need to start thinking about how to take care of it”; “Viewing the earth in its entirety broadened my perspective (…) I have seen the undivided earth from space. When viewed from this perspective, the fighting amongst ourselves makes no sense whatsoever.”

## Conclusion

This article has attempted to remediate Messac’s (1936) long forgotten lament that the insights provided by the Gulliver theme in literature were left underexplored. I have gathered a vast array of scattered reflections, insights and studies on the topic and offered the first cognitively oriented overview of the theme. A review of classical and lesser-known literary works involving altered body–environment scaling has allowed highlighting the manifold components of human’s psychology of size. Based on these observations, I have proposed the first taxonomy of the Gulliver theme and delineated its main features in the perception, action, bodily, affective, cognitive, and social domains. Although derived from purely imaginative works, I suggest that these insights provide a remarkable foundation for the science of architectonic embodiment, as they encompass the full range of human experience that could (or should) be considered by architects and urban designers with respect to the size factor. To reinforce this foundation and favor a cross-disciplinary approach to architectonic embodiment, I have also reviewed available experimental data relevant to the psychological features of the Gulliver theme. Although mostly indirect at the moment, such evidence, assembled here for the first time, already indicates that considering body–environment scaling and its effects on the human mind is of foremost relevance and importance for architecture and related fields.

The current paper also contributes to the growing field of cognitive literary studies (e.g., Richardson and Steen, 2002; Pinker, 2007; Mar and Oatley, 2008). I suggest that exploring the cognitive foundations of specific genres and subgenres, as well as transversal themes such as the Gulliver theme, will enrich historical, cultural, and linguistic approaches to literature. More provocatively, I have suggested elsewhere that common neurocognitive features underlie psychological experience and pathology, literary imagination and creation, and the enjoyment and understanding of literature by readers (Dieguez, 2013; Dieguez and Annoni, 2013b). The successful confluence of such mechanisms could explain the prominence of specific themes throughout literary history, such as the topic of the double and of amnesia, and I suggest it would also explain why the Gulliver theme, as it were an unlikely idea when taken at face value, survived and featured so prominently in so many stories and tales.

## Author Contributions

SD initiated, researched, and wrote the article.

## Conflict of Interest Statement

The author declares that the research was conducted in the absence of any commercial or financial relationships that could be construed as a potential conflict of interest.

## Footnotes

1 Other overviews of the topic can be found in the online Encyclopedia of Science-Fiction (http://www.sf-encyclopedia.com, see for instance the entries “Great and small” and “Miniaturization”); for more recent treatments in popular culture, see also http://tvtropes.org and the entries “Incredible Shrinking Man,” “Sizeshifter,” “Fantastic Voyage plot,” and the like.

2 Together with Robert Hooke, Leeuwenhoek was the foremost popularizer and early user of the microscope as a scientific tool.

3 Comics and Science-Fiction magazines have depicted a flurry of superheros displaying the ability to shrink or grow, highlighting Schuhl’s (1952) insight that the Gulliver theme is indeed “one of the favorite illusions of our imagination.” Characters include The Atom, Ant-Man, Shrinking Violet, Atom Smasher, Doll Man, Micro-Might, and many more.

4 These issues are those that have most occupied commentators of the Gulliver theme, underscoring countless scientific errors, inaccuracies, contradictions and impossibilities in these stories (Haldane, 1926; Messac, 1936; Moog, 1948; Koyré, 1950; Schuhl, 1952; Went, 1968; Bonner, 2006; Rey, 2014). Planets, humans, insects, and microbes as they currently are, it seems, are just the “right size” for existing and for their “purposes.” Any significant size-alteration without correlative changes in surface, structure, shape and complexity would mean inevitable collapse (perhaps a reassuring thought for those with spider phobia). Because it provides such a convenient *reductio ad absurdum* of biological and physical principles, the Gulliver theme is much appreciated in the scientific literature on allometry and development.

5 For a sub-taxonomy of voyages inside the body, see Brodesco (2011).
